# The natural history of progressive fibrosing interstitial lung diseases

**DOI:** 10.1186/s12931-019-1022-1

**Published:** 2019-03-14

**Authors:** Martin Kolb, Martina Vašáková

**Affiliations:** 1 McMaster University and St. Joseph’s Healthcare, 50 Charlton Avenue East, Hamilton, Ontario L8N 4A6 Canada; 2 Department of Respiratory Medicine, Thomayer Hospital, Videnska 800, 14059 Prague, Czech Republic

**Keywords:** Pulmonary fibrosis, Connective tissue diseases, Rheumatic diseases, Systemic sclerosis, Vital capacity, Mortality

## Abstract

A proportion of patients with certain types of interstitial lung disease (ILD), including chronic hypersensitivity pneumonitis and ILDs associated with autoimmune diseases, develop a progressive fibrosing phenotype that shows similarities in clinical course to idiopathic pulmonary fibrosis. Irrespective of the clinical diagnosis, these progressive fibrosing ILDs show commonalities in the underlying pathogenetic mechanisms that drive a self-sustaining process of pulmonary fibrosis. The natural history of progressive fibrosing ILDs is characterized by decline in lung function, worsening of symptoms and health-related quality of life, and early mortality. Greater impairment in forced vital capacity or diffusion capacity of the lungs for carbon monoxide, and a greater extent of fibrotic changes on a computed tomography scan, are predictors of mortality in patients with fibrosing ILDs. However, the course of these diseases is heterogenous and cannot accurately be predicted for an individual patient. Data from ongoing clinical trials and patient registries will provide a better understanding of the clinical course and impact of progressive fibrosing ILDs.

## Background

**Fig. 1:**
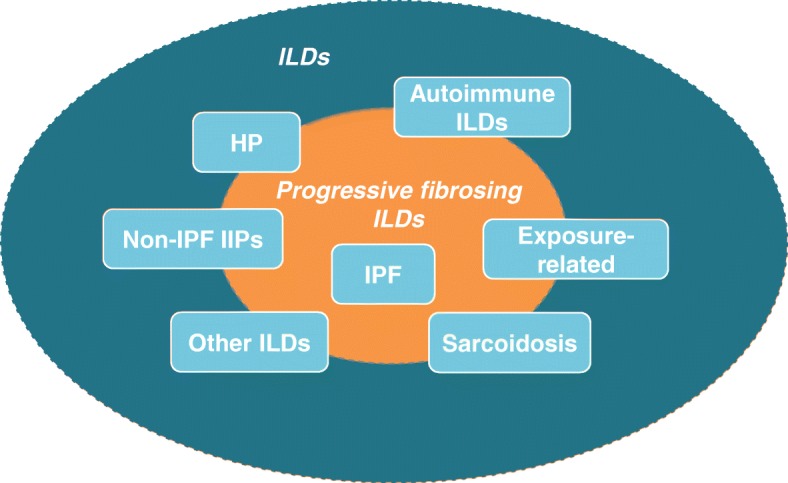
Types of interstitial lung disease associated with a risk of developing a progressive fibrosing phenotype. HP, hypersensitivity pneumonitis; ILD, interstitial lung disease; IIP, idiopathic interstitial pneumonia; IPF, idiopathic pulmonary fibrosis

Interstitial lung diseases (ILDs) encompass a large and varied group of parenchymal lung disorders, including diseases of unknown cause known as the idiopathic interstitial pneumonias (IIPs), as well as those associated with other diseases or environmental exposures. The most extensively studied type of ILD is idiopathic pulmonary fibrosis (IPF), which occurs mainly in adults aged over 60 years and is characterized by progressive pulmonary fibrosis, decline in lung function and high mortality [1]. A proportion of patients with other types of ILD also develop a progressive fibrosing phenotype that shows similarities in underlying pathogenetic mechanisms and clinical behavior to IPF [2, 3]. ILDs that may be associated with a progressive fibrosing phenotype include connective tissue disease-related ILDs (CTD-ILDs) such as those related to rheumatoid arthritis (RA-ILD) [4], systemic sclerosis (SSc-ILD) [5], and polymyositis/dermatomyositis [6]; ILD related to chronic sarcoidosis [7]; chronic hypersensitivity pneumonitis (HP) [8]; idiopathic non-specific interstitial pneumonia (iNSIP) [9] and unclassifiable ILD [10] (Fig. 1). The proportion of patients with non-IPF ILDs who develop a progressive fibrosing phenotype is not known, but has been estimated by physicians who manage patients with non-IPF ILDs to be up to 40% [11, 12]. In this review, we will describe the natural history of progressive fibrosing ILDs.

## Pathogenesis of progressive fibrosing ILDs

Some ILDs are primarily considered fibro-proliferative disorders, in which alveolar epithelial injury and fibroblastic proliferation lead to fibrosis, while other ILDs are considered primarily inflammatory disorders in which the pathogenic process shifts to a fibro-proliferative pathway under certain conditions [13, 14]. Regardless of the trigger, fibrosing ILDs show commonalities in the mechanisms involved in their pathogenesis and progression. Repeated chronic epithelial or vascular injuries lead to cell destruction and to unregulated repair [15–17]. Fibroblasts proliferate, migrate from different sources to the site of injury and are activated to become myofibroblasts, which secrete increased amounts of extracellular matrix. This, together with reduced matrix degradation, results in increased tissue stiffness and loss of function of the alveolar tissue [18–22]. Macrophages and lymphocytes are recruited to the site of injury and release pro-fibrotic mediators that further promote fibroblast activation [18]. In a feed-forward loop, the increased lung tissue stiffness further activates and stimulates fibroblasts to drive a self-sustaining process of fibrosis [23, 24]. As an increasing extent of the lung is lost to fibrosis, the volume of the lung is reduced and gas exchange impaired, resulting in worsening breathlessness and capacity for exertion, and ultimately in respiratory failure.

## Lung function decline in progressive fibrosing ILDs

**Fig. 2:**
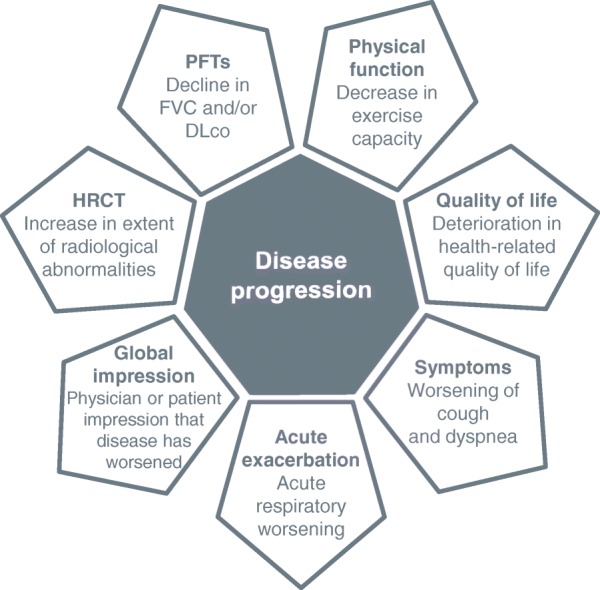
Factors that reflect progression of interstitial lung diseases. DLco, diffusion capacity of the lungs for carbon monoxide; FVC, forced vital capacity; HRCT, high-resolution computed tomography; PFTs, pulmonary function tests

**Fig. 3:**
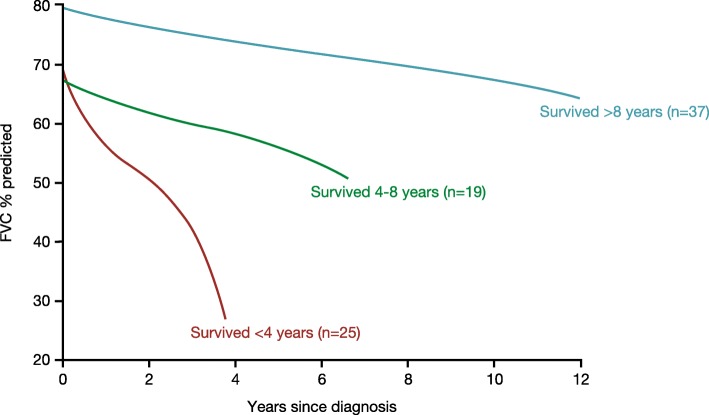
Decline in FVC % predicted in patients with SSc-ILD categorized by survival time from diagnosis of ILD. Adapted from [31]. Reprinted with permission of the American Thoracic Society. Copyright© 2019 American Thoracic Society. Guler SA et al. 2018. Does systemic sclerosis-associated interstitial lung disease burn out? Specific phenotypes of disease progression. Ann Am Thorac Soc 2018;15:1427–1433. *Annals of the American Thoracic Society* is an official journal of the American Thoracic Society

**Fig. 4:**
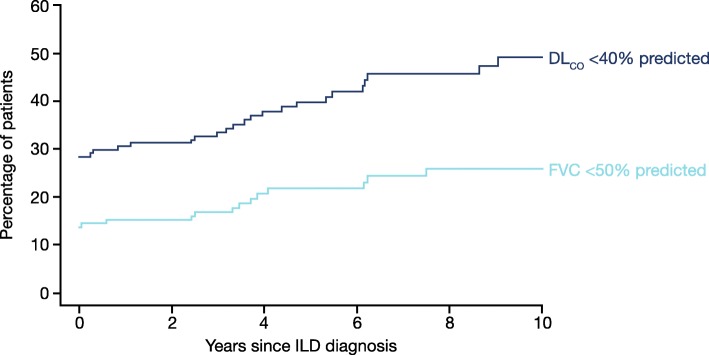
The proportions of patients with FVC < 50% predicted and DLco < 40% predicted in the 10 years from diagnosis of ILD associated with RA. Adapted from [4]

Progression of fibrosing ILD is reflected in a decline in lung function, worsening of symptoms and deterioration in health-related quality of life (Fig. 2) [25–28]. In clinical studies, disease progression is most commonly assessed through measurement of forced vital capacity (FVC) and diffusion capacity of the lungs for carbon monoxide (DLco). The course of lung function decline in patients with IPF is variable, but IPF is, by definition, a progressive disease [1]. Other fibrosing ILDs are also heterogeneous but progressive in their clinical course. In patients with SSc, ILD is more common in patients who have diffuse cutaneous disease or are positive for anti-topoisomerase I antibodies [29]. The risk of development and progression of ILD seems to be greatest in the few years after diagnosis. Among 695 patients with SSc in the European League Against Rheumatism (EULAR) Scleroderma Trials and Research group (EUSTAR) cohort, approximately one third of patients had a DLco < 50% predicted within three years of the onset of Raynaud’s phenomenon [30]. A recent study of 81 patients at a specialized SSc-ILD clinic who were followed for ≥8 years after diagnosis or died during follow-up identified three distinct subgroups with different rates of FVC decline and mortality (Fig. 3). In all the subgroups, FVC decline was largely linear over the long term, but was too variable over the short term to enable recent change to be used to predict future change [31]. Patients with other CTD-ILDs may also suffer rapid loss of lung function after ILD develops. In an analysis of 167 patients with RA-ILD referred to a single tertiary care center, the proportion of patients with FVC < 50% predicted increased from 14% at diagnosis to 22% after 5 years (Fig. 4) [4]. In an analysis of 107 patients with ILD associated with polymyositis/dermatomyositis, 16% had a decline in FVC of ≥10% predicted and/or a decline in DLco of ≥15% predicted over a median follow-up of 34 months, despite treatment [6].

Although some patients with chronic HP experience partial recovery, patients with chronic fibrosing HP may experience rapid disease progression, particularly if the inciting antigen cannot be identified and removed [32, 33]. In a recent longitudinal cohort analysis, the monthly decline in FVC % predicted over 1 year in 119 patients with chronic HP was similar to that of 286 patients with IPF [34]. In patients with stage IV pulmonary sarcoidosis, both increases and decreases in FVC and DLco may be observed over time as the disease relapses and remits [35]. Little is known about the clinical course of fibrotic iNSIP, but it seems to progress more slowly than other forms of fibrosing ILD [36].

## Acute exacerbations of fibrosing ILDs

Acute exacerbations - episodes of rapid respiratory worsening accompanied by evidence of new ground glass opacities on HRCT - are a common feature of the natural history of IPF, believed to occur in 5–10% of patients per year [37]. Acute exacerbations of IPF may be idiopathic or triggered by an insult such as infection or aspiration. Irrespective of their cause, acute exacerbations of IPF are associated with very high morbidity and mortality, with a median post-event survival of only 3 to 4 months [37]. Far fewer data are available on acute exacerbations of fibrosing ILDs other than IPF, and most come from retrospective reviews of medical records. However, it appears that acute exacerbations do occur in patients with fibrosing ILDs other than IPF and are often fatal. An analysis of medical records from 84 patients with RA-ILD from two tertiary referral centers found that 14 patients (17%) had an idiopathic acute exacerbation over a median follow-up of 33 months, and 13 of those 14 patients died within 1.5 months of the event [38]. A study of 51 patients with RA-ILD at another center found that 22% had an acute exacerbation over a follow-up period of about 8 years [39]. In patients with SSc-ILD, acute exacerbations appear to be more common in patients with a usual interstitial pneumonia (UIP) pattern on computed tomography or histology [40, 41]. A study of 100 patients with chronic HP found that 14 patients were hospitalized for an acute exacerbation over a 2-year period, of whom 11 died within one month; patients with a UIP pattern on histology were more likely to experience acute exacerbations than those with other patterns [42].

## Mortality in patients with progressing fibrosing ILDs

Progressive fibrosing ILDs are associated with high mortality. In patients with IPF who are not receiving an antifibrotic therapy, median post-diagnosis survival is 3–4 years [43–45]. ILD is one of the leading causes of death in patients with SSc [46]. Estimates of survival time vary depending on the population studied, but a recent study of patients at a specialized SSc-ILD clinic found that median survival was 11.2 years from the date of the first HRCT showing evidence of ILD [31]. RA-ILD is also associated with high mortality, particularly in patients with a UIP pattern on HRCT [47–49]. A retrospective analysis of 82 patients with RA-ILD at two tertiary referral centers found a median survival time of 5 years from the initial clinic visit (3.2 years in patients with a UIP pattern on HRCT) [48]. Patients with features consistent with the research construct known as interstitial pneumonia with autoimmune features (IPAF), i.e.*,* who have interstitial pneumonia with features suggestive of an autoimmune disease but do not meet criteria for a defined disease [50] appear to have a mortality rate that is intermediate between patients with IPF and CTD-ILDs [51]. Fibrotic HP has a poor prognosis. A recent analysis of US claims data found that only 58% of patients with fibrotic HP were still alive approximately 7 years after diagnosis [52]. Fibrotic iNSIP appears to have a better prognosis than other forms of progressive fibrosing ILD: considering only disease-related death, 5-year survival is approximately 75% [53]. Assessment of the mortality associated with unclassifiable IIP is hampered by the wide variability in case definition across studies, but a systematic literature review found estimates for 5-year survival in patients with unclassifiable ILD, based on four studies of 46 to 70% [54].

## Predictors of disease progression in patients with fibrosing ILDs

Studies in patients with progressive fibrosing ILDs have identified several factors that predict mortality, but these need to be interpreted carefully given the variation in the methodology used and the retrospective nature of most of the studies. Lower FVC is an established predictor of mortality in patients with progressive fibrosing ILDs, as evidenced by numerous studies spanning IPF [55–57], RA-ILD [4, 49], SSc-ILD [58–60], chronic HP [61, 62] and fibrotic iNSIP [53]. The same is true of DLco [55, 56, 60, 63–65], although this is harder to assess in multi-center studies due to a lack of standardization in its measurement. A decline in FVC > 10% predicted is another well-established predictor of mortality [49, 56, 62, 65], but smaller declines in FVC have also been shown to be associated with a worse prognosis, at least in patients with IPF [66–68]. Further, it is important to bear in mind that “small” annual declines in FVC may become substantial when summed over a period of years. This is particularly relevant when considering ILDs with a relatively early age of onset.

Composite scoring systems have been developed to predict mortality in patients with progressive fibrosing ILDs. One of the most widely used is the GAP (gender, age, physiology) model, which was developed to predict mortality in patients with IPF based on gender, age, FVC % predicted and DLco % predicted [69], and has since been shown to predict mortality in patients with RA-ILD [70], SSc-ILD [71], unclassifiable ILD [72] and a mixed cohort [73]. Composite scoring systems have also been developed specifically for prediction of progression of SSc-ILD [74, 75]. Unfortunately, none of the scoring systems developed to date provides an accurate prediction of the way that ILD will progress in an individual patient, creating challenges for therapeutic decision-making and patient counselling. Recently a cluster analysis in a cohort of 770 patients with diverse ILDs (IPF, CTD-ILDs, chronic HP, IPAF) identified four phenotypic groups based on factors such as age, race, smoking, and radiological features that differed in FVC decline and survival [34]. Whilst interesting from an academic perspective, the use of such groupings in clinical practice remains to be established.

There is increasing interest in the use of radiological markers as predictors of disease progression in patients with fibrosing ILDs. A greater extent of fibrotic changes on HRCT is known to be predictive of mortality. This has been demonstrated in several studies in IPF [76], RA-ILD [48, 77], SSc-ILD [5, 58], chronic HP [61, 78], pulmonary sarcoidosis [79] and unclassifiable ILD [63]. In addition, specific radiological features such as honeycombing and traction bronchiectasis have been associated with worse prognosis [33, 80]. However, the use of radiological scoring systems as predictors of prognosis in clinical practice is hampered by subjectivity and large inter-observer variability in reading HRCT scans, and will probably not be widely implemented until validated, low-cost, automated scoring systems are available [81].

Several blood biomarkers have been investigated as predictors of disease progression in patients with fibrosing ILDs. These include Krebs von den Lungen-6 protein (KL-6), which has been associated with a higher rate of disease progression in patients with IPF and CTD-ILDs [82–85], and surfactant protein-D (SP-D) [86, 87]. Several studies have shown that levels of matrix metalloproteinase-7 (MMP-7), an enzyme involved in remodeling of the extracellular matrix, are negatively correlated with lung function and survival in patients with IPF [88–91]. Protein fragments generated by breakdown of the extracellular matrix have also been investigated as predictors of disease progression [92, 93]. To date, however, no serum biomarker has been shown to be a sufficiently robust prognostic marker to justify its use in clinical practice, although KL-6 is routinely used at some ILD centers in Japan.

Genetic mutations and telomere length have also been investigated as predictors of disease progression in patients with fibrosing ILDs. In patients with IPF, rs35705950, the minor allele of a single nucleotide polymorphism (SNP) in *MUC5B,* a gene encoding a component of mucus secretions, has been associated with improved survival [94] while rs5743890, the minor allele of an SNP of *TOLLIP* (toll interacting protein), has been associated with worse survival [95]. However, in patients with chronic HP, neither of these alleles was associated with survival [96]. In patients with features consistent with IPAF, *MUC5B* rs35705950 was associated with worse survival, while there was no association between TOLLIP genotype and survival [97]. Recently, MUC5B rs35705950 was associated with the development of RA-ILD [98]. In addition, mutations in several genes related to telomere maintenance, such as telomerase reverse transcriptase (TERT) and telomerase RNA component (TERC), have been identified in patients with IPF [99], and patients with short telomeres have reduced survival [100, 101]. Short telomere length has also been associated with worse survival in patients with features consistent with IPAF [97] and in patients with chronic HP [96]. Variants in telomere-related genes have been identified in patients with RA-ILD [102] and SSc-ILD [103], but further investigation is needed into the association between these variants and the development and progression of ILD.

## Further research into the natural history of progressive fibrosing ILDs

Data from clinical trials of investigational therapies made a large contribution to our understanding of the clinical course of IPF [104]. Ongoing large clinical trials conducted in patients with non-IPF fibrosing ILDs [105–108] will provide valuable insights into the progression of these diseases in well-characterized populations. In addition, the natural history of fibrosing ILDs is being investigated in many patient registries including the Pulmonary Fibrosis Foundation Patient Registry [109] and IPF-PRO/ILD-PRO Registry [110] in the USA, the Canadian Registry for Pulmonary Fibrosis (CARE-PF) [111], the INSIGHTS-IPF [112] and EXCITING-ILD [113] registries in Germany, and the EMPIRE registry in central and Eastern Europe [60]. These and other registries will provide valuable information on the course of fibrosing ILDs diagnosed and managed in clinical practice, including their impact on lung function, patient-reported outcomes, hospitalizations and mortality. In addition, further research is needed into the impact that comorbidities such as pulmonary hypertension and ischemic heart disease have on outcomes in patients with ILDs [114].

## Conclusions

A proportion of patients with fibrosing ILDs develop a progressive fibrosing phenotype that is similar to IPF in clinical behavior and in many of the underlying pathogenetic mechanisms that drive a self-sustaining process of pulmonary fibrosis. The natural history of progressive fibrosing ILDs is characterized by deterioration in lung function, worsening dyspnea and high mortality. A greater extent of fibrosis on HRCT and decline in lung function are predictors of mortality, but the course of disease for an individual patient cannot accurately be predicted using the currently available tools. Ongoing research will provide a better understanding of the clinical course of progressive fibrosing ILDs and their impact on patients.

## Abbreviations

CTD-ILDs: Connective tissue disease-related ILDs
DLco: Diffusion capacity of the lungs for carbon monoxide
EULAR: European League Against Rheumatism
EUSTAR: EULAR Scleroderma Trials and Research group
FVC: Forced vital capacity
HP: Hypersensitivity pneumonitis
ILD: Interstitial lung disease
iNSIP: Idiopathic non-specific interstitial pneumonia
IPAF: Interstitial pneumonia with autoimmune features
IPF: Idiopathic pulmonary fibrosis
MMP-7: Matrix metalloproteinase-7
RA-ILD: Rheumatoid arthritis ILD
SSc-ILD: Systemic sclerosis ILD
UIP: Usual interstitial pneumonia
